# Assessment of glycaemic status in adult hospital patients for the detection of undiagnosed diabetes mellitus: A systematic review

**DOI:** 10.1111/dme.14777

**Published:** 2022-01-05

**Authors:** Tabitha D. Thornton‐Swan, Laura C. Armitage, Aisling M. Curtis, Andrew J. Farmer

**Affiliations:** ^1^ Exeter College University of Oxford Oxford UK; ^2^ Clinical Medical School University of Oxford Oxford UK; ^3^ Nuffield Department of Primary Care Health Sciences University of Oxford Oxford UK; ^4^ Green Templeton College University of Oxford Oxford UK

**Keywords:** blood glucose, diabetes mellitus, diagnostic tests, glycated haemoglobin A, hyperglycaemia, mass screening, routine

## Abstract

**Aim:**

In‐hospital blood glucose testing is commonplace, particularly in acute care. In‐hospital screening for hyperglycaemia may present a valuable opportunity for early diabetes diagnosis by identifying at‐risk individuals. This systematic review investigates the extent to which random blood glucose testing in acute and inpatient hospital settings predicts undiagnosed diabetes.

**Methods:**

Two databases were systematically searched for studies in which adult patients received an in‐hospital random blood glucose test, followed by a diagnostic HbA1c test. The primary outcome was the proportion of hyperglycaemic individuals diagnosed with diabetes by HbA1c.

**Results:**

A total of 3245 unique citations were identified, and 12 were eligible for inclusion. Ten different blood glucose thresholds, ranging from 5.5 to 11.1 mmol/L, were used to detect hyperglycaemia, indicating that there is no consistent clinical definition for hyperglycaemia. The proportion of participants with hyperglycaemia in each study ranged from 3.3% to 62.1%, with a median (Q_1_, Q_3_) of 34.5% (5.95%, 61.1%). The proportion of hyperglycaemic participants found to have a diabetes‐range HbA1c varied from 4.1% to 90%, with a median (Q_1_, Q_3_) of 18.9% (11.5%, 61.1%). Meta‐analysis was not possible due to substantial heterogeneity between study protocols.

**Conclusions:**

All studies consistently identified a proportion of hyperglycaemic hospital patients as having a diabetes‐range HbA1c, showing that in‐hospital blood glucose screening can facilitate diabetes diagnosis. The proportion of hyperglycaemic participants with undiagnosed diabetes varied substantially, indicating a need for further research and consistency in defining in‐hospital hyperglycaemia. This may aid the development of a standardised screening protocol to identify people with possible undiagnosed diabetes.

**Novelty Statement**

**What is already known?**

- Undiagnosed diabetes is a global public health concern, showing that current diagnostic methods are insufficient
- Random blood glucose is frequently tested as part of routine care for individuals admitted to hospital

**What this study has found?**

- A consistent clinical definition for hyperglycaemia is lacking
- In‐hospital hyperglycaemia can be predictive of undiagnosed diabetes

**What are the implications of the study?**

- Further research is needed to identify the in‐hospital blood glucose threshold with optimal sensitivity and specificity to guide selection for subsequent diagnostic testing to detect undiagnosed diabetes
- Consistency in defining hyperglycaemia in the field is required so that studies may be compared for reproducibility of findings and to permit clinically important outcomes to be translated into the clinical setting

## 1 INTRODUCTION

The International Diabetes Federation estimates that 463 million adults aged 20–79 currently have diabetes mellitus (DM). This accounts for 9.3% of the global adult population, a proportion predicted to reach 10.2% by 2030.^1^ Diabetes is a chronic metabolic condition caused by insufficient production or function of insulin, resulting in impaired glycaemic control and hyperglycaemia. In 2019, 50.1% of the world's adult population with diabetes were undiagnosed.^1^ Current UK diagnostic methods for diabetes, consisting of NHS Health Checks and opportunistic blood testing in primary care, are insufficient, as approximately 850,000 people in the United Kingdom are estimated to have undiagnosed Type 2 diabetes.^2^ Untreated diabetes is associated with an increased risk of complications^3^ such as neuropathy, retinopathy, cardiovascular and kidney diseases,^4^ evidence of which is found in 50% of people with newly diagnosed Type 2 diabetes.^5^ Early detection of diabetes is, therefore, vital to prevent unnecessary mortality and morbidity and to reduce the burden on the population, society and healthcare services.

The World Health Organisation's (WHO) diagnostic criteria for diabetes are a fasting plasma glucose (FPG) ≥7.0 mmol/L or a 2‐hour plasma glucose ≥11.1 mmol/L during a 75 g oral glucose tolerance test (OGTT).^6^ A random plasma glucose measurement of 11.1 mmol/L in patients presenting with classic symptoms of hyperglycaemia is also considered diagnostic.^6^, ^7^ Since 2011, WHO diagnostic guidelines have included a glycated haemoglobin assay (HbA1c), which defines diabetes according to a threshold of ≥48 mmol/mol (≥6.5%) on two occasions.^8^ Both WHO and NICE guidelines state that two HbA1c, RPG or FPG measurements within the diagnostic range are required to diagnose an asymptomatic individual with diabetes.^7^, ^8^ HbA1c has a strong positive correlation with average plasma glucose 8–12 weeks prior to testing^9^ and does not require fasting, so it can be used for diagnostic purposes and monitoring glycaemic control in people with diabetes.^7^, ^10^, ^11^, ^12^ Testing can also identify non‐diabetic hyperglycaemia; an HbA1c between 42 mmol/mol (6.0%) and 47 mmol/mol (6.4%) indicating increased risk of developing diabetes later in life.^7^ Identifying non‐diabetic hyperglycaemia can allow for early intervention and lifestyle changes to reduce risk,^13^ strategies that are now coordinated through the NHS Diabetes Prevention Programme.^14^ Implementation of an in‐hospital strategy for identifying individuals at high‐risk for diabetes may have broad reach and application, given there are around 17 million annual admissions to hospitals in England alone^15^ and an estimated 11% of the English population have non‐diabetic hyperglycaemia.^16^

In‐hospital screening for hyperglycaemia may allow cost‐effective identification of individuals with undiagnosed diabetes. The American Diabetes Association (ADA) now recommends that HbA1c tests should be performed on all individuals admitted to hospital with a blood glucose greater than 7.8 mmol/L.^17^ Blood glucose tests are easy, inexpensive and frequently used in hospital,^18^ and hyperglycaemia is common in both acute care and inpatient populations.^18^, ^19^, ^20^ A recent clinical audit conducted over a year in a large UK teaching hospital by Ghosh et al.^18^ found that 86% of individuals admitted as an emergency with no prior diabetes diagnosis had a glucose measurement taken. 21% of hospital admissions with no prior diabetes coding had recorded random blood glucose measurements above 7.8 mmol/L. Inpatient hyperglycaemia is not always indicative of diabetes; blood glucose can vary depending on food intake, clinical treatment and acute stress. However, in‐hospital screening for hyperglycaemia using random blood glucose may identify a population to be targeted for subsequent diagnostic testing and could present a window of opportunity for early diagnosis.

In this review, we investigate the extent to which elevated random blood glucose in the hospital setting can detect previously undiagnosed diabetes in adults. The purpose of this review is to identify an optimum threshold value for in‐hospital random blood glucose, above which people are likely to receive a diabetes diagnosis with HbA1c.

## 2 METHODS

The Preferred Reporting Items for Systematic Reviews and Meta‐Analysis Diagnostic Test Accuracy (PRISMA‐DTA) guidelines^21^ were followed. The protocol for this systematic review was registered on the International Prospective Register of Systematic Reviews (PROSPERO: registration number CRD42021226227) before commencement of data extraction.

### 2.1 Eligibility criteria

Retrospective or prospective cohort studies were included if the glycaemic status of adults was evaluated during hospital admission with a random blood glucose test (index test) and an HbA1c test (reference test) performed for individuals whose random blood glucose exceeded a predefined study threshold.

Studies were eligible if the participants studied met all of the following criteria:

- Aged 18 and over
- No pre‐existing diagnosis of DM
- Initial admission to a surgical/medical ward or attendance at the emergency department (ED)
- Reason for initial admission was not due to diabetes, acute coronary syndrome or stroke
- Study setting was not an intensive care unit (ICU)
- Not pregnant

With reference to criterion II, studies were required to have screened their study population for the presence of a pre‐existing diagnosis of DM and to report reference test diagnostic data for a cohort with no pre‐existing diabetes. Studies were permitted for inclusion if they reported data separately for cohorts with and without pre‐existing DM. Studies recruiting only ICU or acute cardiac patient populations were excluded during the screening of citations because stress hyperglycaemia is more common in these groups compared with a typical hospital patient population.^22^ Elevated random blood glucose measurements are, therefore, much less likely to reflect chronic hyperglycaemia in these populations, resulting in a high rate of false positives.

The primary outcome was the number of study participants with a random blood glucose level above the study‐defined threshold who received a new diagnosis of diabetes on HbA1c testing. Other reported diagnostic metrics, including sensitivity and specificity of random blood glucose for predicting diabetes, were recorded as additional outcomes if available.

### 2.2 Search strategy

PubMed and EMBASE were searched for relevant articles from the inception of each database until 11 January 2021. The search strategy was developed in collaboration with a medical information specialist. No language, country or date restrictions were applied. The search terms were as follows:

1. in‐hospital OR hospitalised OR inpatient OR “emergency department” OR hospitalized

AND

- hyperglycaemia OR "raised glucose” OR glucose OR hyperglycemia

AND

- "formal assessment" OR diagnosis OR follow‐up OR outpatient OR community

AND

- "diabetes mellitus" OR HbA1c OR “glycated haemoglobin" OR "Haemoglobin A1c" OR “glycated hemoglobin” OR “Hemoglobin A1c”

AND

- undiagnosed OR “no prior history" OR "asymptomatic" OR "without known diabetes" OR "without a diagnosis"

Results included both published manuscripts and conference abstracts. Full search strategies are provided in the appendix.

### 2.3 Study selection

All returned citations from the database searches were imported into Rayyan, a systematic review manager application.^23^ Rayyan was then used to remove any duplicate citations returned by the database searches. Each citation was then independently screened by title and abstract by two of three reviewers (TTS, LA and AC). Conflicts were resolved through adjudication with a third reviewer (either TTS, LA or AC). Those citations assessed as being potentially eligible articles were then screened by full text. Each full text was screened by two independent reviewers (TTS and LA), and any conflicts were resolved through adjudication with a third reviewer (AF). Authors of any relevant conference abstracts were contacted by email for additional data required to assess eligibility during the screening process.

### 2.4 Data extraction and synthesis

Data extraction was conducted by two independent reviewers (TTS and LA) using a custom Microsoft Excel data extraction form. Where available, extracted data included study and cohort characteristics (publication year, study design, study nation, hospital setting and department, enrolment period, size of the screened and study populations, age, gender, ethnicity and exclusion criteria, including the number excluded due to a prior diabetes diagnosis), as well as data about the index and diagnostic tests deployed.

The following index test data were extracted from each paper:

- Number of screened participants who had a random blood glucose test
- Type of blood sample used for index test (capillary, venous)
- Timing of index test during hospital admission
- Threshold used to define hyperglycaemic status
- Thresholds to subclassify severity of hyperglycaemia
- Number of study participants with a glucose level above a predefined threshold

The following outcome data relating to the reference test were extracted from each paper:

- Timing of reference diagnostic HbA1c test (during or post‐hospital admission)
- HbA1c threshold used to diagnose diabetes
- Number of hyperglycaemic study participants who received an HbA1c test
- Number of participants with a diagnosis of diabetes on HbA1c testing

Where available, sensitivity and specificity data were extracted for each index test, as well as any other data evaluating the performance of the applied random glucose thresholds for detecting undiagnosed diabetes. Averages are given as median (Q_1_, Q_3_) unless specified otherwise.

### 2.5 Risk of bias and applicability

Potential bias and applicability of included studies were assessed independently by two reviewers (TTS and LA) using QUADAS‐2 (Quality Assessment of Diagnostic Accuracy Studies^24^). Disagreements were adjudicated by a third reviewer (AF). QUADAS‐2 assesses risk of bias and applicability with respect to four domains:

- patient selection
- index test
- reference standard
- flow and timing

Domain (iv) is only assessed for bias. Specific questions are recommended for the assessment of each domain, two of which were not relevant for this review, as they assess whether the index and diagnostic tests were blinded; both HbA1c and random blood glucose have numerical thresholds, so knowledge of one result would not bias interpretation of the other. Risk of bias and concerns regarding applicability were assessed as low, high or unclear for each domain. No quantitative overall score was calculated, but a study assessed as low for all domains was considered at low risk of bias or applicability.

## 3 RESULTS

**FIGURE 1:**
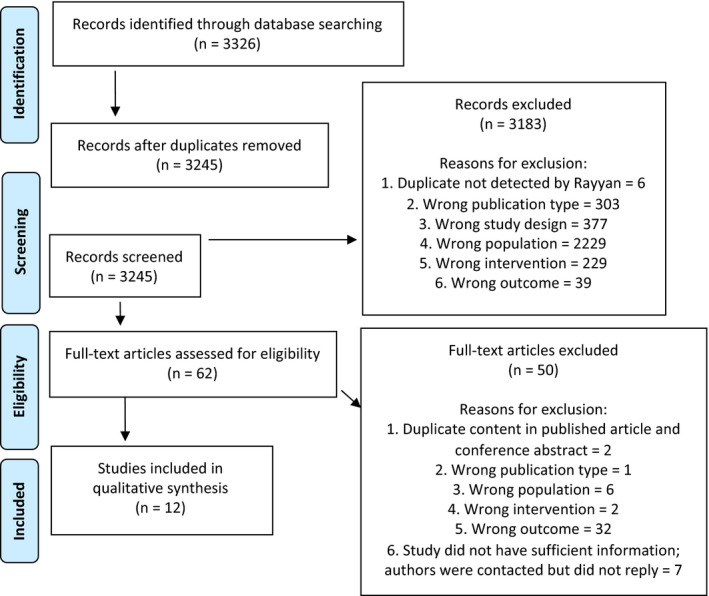
PRISMA flow diagram for study selection

The database search returned 3326 citations and 3245 unique publications after duplicates were removed. Sixty‐two studies (1.9%) were selected for full text review after title and abstract screening. Of these, 12 (19.3%) met inclusion criteria. The screening process and reasons for exclusion are shown in Figure 1.

Conflicts between reviewers regarding reasons for non‐eligibility were resolved by applying a hierarchy of reasons for exclusion as reported in Figure 1. The most common reason for exclusion at full text screening was ‘wrong outcome’; owing to either a lack of formal assessment or reference test for hyperglycaemic study participants (*n* = 26), or the use of a diagnostic test other than HbA1c (*n* = 6). The authors of eight potentially eligible citations were contacted by email for further information, six of which were for conference abstracts. One author provided sufficient information to permit inclusion of their study.^25^

Across the 12 included studies, 25,987 individuals had a random blood glucose test. Follow‐up HbA1c test results were available for 5517 participants (21.2%) who were classified as hyperglycaemic using a study‐defined blood glucose threshold. The study characteristics are reported in Table 1.

**TABLE 1:** Characteristics of included studies

Primary author	Study details		Cohort characteristics											
	Year of publication	Study design	Study nation	Ethnicity	Type of hospital setting	Hospital department or specialty caring for participant at index admission	Period of enrolment*	Size of screened population	Number of eligible patients	Size of study population	Age	Gender (% male)	Important exclusions from the cohort	Proportion excluded due to a prior diagnosis of diabetes
Berger	2018	Case control, prospective	United States	Unknown	Tertiary academic	ED	January 2014 and November 2017	889	487	332	Unknown	Unknown	<18 y/o, existing diabetes diagnosis, pregnancy, inability to consent	Unknown
Biesman‐Simons	2019	Multicentre prospective	South Africa	Unknown	6 public hospitals	Surgical ward (elective patients)	16 October to 20 October 2017	Unknown	391	379	Mean 50.6 (SD 16.5)	36.4	Refusal/inability to consent, emergency/cardiac surgery, pregnancy, <18 y/o	16.1% (61/379)
Epa	2020	Nested cohort^†^	Australia	Unknown	Tertiary academic	ED	1 July to 31 December 2015	Unknown	16,268	16,268	Mean 58.6 (SD 21.5)	57	<18 y/o, blood sample already being tested available	9.4% (1,534/16,268)
Ginde	2008	Prospective	United States	Non‐Hispanic White 80%, Non‐Hispanic African American 8%, Hispanic 5%, Non‐Hispanic Other 6%.	Urban academic	ED	8x24 hr periods, 4 weekdays, 4 weekend days, during April to August 2007	1,611	789	355	53% 18–44, 33% 45–65, 47% ≥65	53	Prior diabetes diagnosis (except gestational diabetes only), high acuity/distress, altered mentation/acute psychiatric illness, history of possible sexual assault	13.5% (48/355)
Hng	2016	Prospective	Australia	Unknown	Urban public	ED	6‐week enrolment period, dates not provided	4,580	2,652	2,652	Unknown	52.7	Pregnant, <16 y/o	67.8% (330/487)
Jelinek	2010	Prospective	Australia	Unknown	Tertiary	ED	Mix of mornings, afternoons, and evenings across 7‐day week, dates not provided	24,081	725	590	Median 53	50.6	Inability to provide consent/speak English, pregnancy, high dependence on medical care, receipt of glucose intervention in ED	18.6% (135/725)
Karakonstantis	2019	Prospective	Greece	Unknown	Community	Internal medicine	October 2017 to April 2018	463	69	55	Median 78 (IQR 65–85)	Unknown	Prior diabetes diagnosis, low admission glucose <100 mg/dl, any condition affecting HbA1c e.g. known haemoglobinopathies, recent blood loss, significant anaemia (Hb <10), red blood cell transfusion within 6 months of admission, significant kidney disease or EPO, >65 y/o with very poor health or end‐stage disease on palliative care, pregnancy, admission due to DKA or hyperglycaemic hyperosmolar state, HbA1c measurement within 3 months of admission	27.6% (128/463)
McNaughton	2015	Prospective	Guyana	Afro‐Guyanese 36.9%, American‐Indian 6.8%, Indo‐Guyanese 36.9%, Mixed 19.4%	Tertiary public	ED	May 21 to August 7 2012 during daytime hours	1,010	270	228	Median 43 (IQR 38–53)	46	Pregnancy, patients <30 y/o, emergency patients, medical/psychological unsuitability (e.g. suspected sexual assault patients, severe pain, intoxication, active bleeding), patients referred for hyperglycaemia/receiving IV glucose	Unknown
O'Sullivan	2014	Retrospective	Ireland	Unknown	Academic	Medical and surgical wards including ED	9‐day period in June 2009	262	140	126	Median 70 (range 19–96)	54	Medical notes unavailable, patients outside general acute ward, <18 y/o	73.0% (92/126)
Silverman	2006	Prospective	United States	White 60%, African‐American 17%, Asian 8%, Caribbean/ Guyanese 9%, Hispanic 6%, Other 1%	Academic	ED	1 May 2003 to 5th Feb 2004	Unknown	Unknown	541	Mean 59.7 (SD 18.5)	46	Prior diabetes diagnosis or history of hyperglycaemia (including during pregnancy), polydipsia/polyuria, ED referral due to hyperglycaemia, systemic corticosteroid use in month prior to admission, IV infusion of glucose, glucagon or epinephrine before testing, acute trauma, pregnancy, inability to consent/speak English	Unknown
Valentine	2011	Prospective	Australia	White 92.9%, ATSI 1.2%, Asian 0.9%, Other 1.1%, Unknown 3.9%	Tertiary academic	All adult admissions	1 April to 30 June 2009	4,691	3,873	2,672	Mean 63.8 (SD 19.6)	52.4	Pregnancy	11.7% (312/2,672)
Wexler	2008	Prospective, patients with abnormal HbA1c have RBG measured	United States	White 86%	Acute care general	All adult admissions	11 days (weekdays and weekends) of July and August 2006	945	695	695	Mean 67 (SD 15)	42	Pregnancy, admission for observation only, blood sample unavailable	18.0% (170/945)

* Data regarding period of enrolment is detailed as provided in the manuscripts of the primary studies; this was not provided in a uniform or standardised format and for several studies the dates of enrolment were not available.

† In this study, a sample of 200 hyperglycaemic participants without known diabetes were selected for further study. These participants were chosen at random from the group of 844 subjects with hyperglycaemia and no known diabetes diagnosis. Further details are provided in Table 3.

Four studies took place in the United States,^25^, ^26^, ^27^, ^28^ four in Australia^29^, ^30^, ^31^, ^32^ and one each in South Africa,^33^ Ireland,^34^ Greece^35^ and Guyana.^36^ Ethnicity was not reported for seven out of 12 studies.^25^, ^29^, ^30^, ^31^, ^33^, ^34^, ^35^ Out of five studies reporting participants’ mean age,^26^, ^27^, ^29^, ^32^, ^33^ the highest was 67 years,^27^ and the lowest was 50.6 years.^33^ Four publications reported median age,^31^, ^34^, ^35^, ^36^ and two gave no information.^25^, ^30^ All but two studies^25^, ^35^ reported the sex of study participants; the average proportion of men was 49.0%.

The majority of studies were based in tertiary hospitals (*n* = 5) and/or academic hospitals (*n* = 6).^25^, ^26^, ^28^, ^29^, ^31^, ^32^, ^34^, ^36^ Four were set in public or community hospitals,^30^, ^33^, ^35^, ^36^ and one in an acute care general hospital.^27^ Out of 12 studies, seven included only ED patient populations,^25^, ^26^, ^28^, ^29^, ^30^, ^31^, ^36^ two screened all hospital admissions^27^, ^32^ and three included individuals from a combination of ED, surgical and/or medical wards.^33^, ^34^, ^35^ Exclusion criteria were generally consistent, although notable exceptions are discussed below. Pregnancy, an existing diabetes diagnosis, age <18 years, and emergency and trauma status were common reasons for exclusion. The proportion of individuals excluded due to a prior diabetes diagnosis ranged from 9.4%^29^ to 73%.^34^

### 3.1 Risk of bias and applicability

The risk of bias and applicability assessment using QUADAS‐2 is shown in Table 2. Overall, only one study was low risk of bias and no concerns regarding applicability,^32^ and two were low risk for either bias or applicability.^29^, ^34^ Six studies were considered at high risk of bias for patient selection. One used extensive exclusion criteria that may limit generalisability of the results to the real‐world setting,^35^ whereas the other five recruited study populations by convenience sampling.^25^, ^26^, ^31^, ^33^, ^36^ One of these^25^ was a case control study, and for the purposes of data extraction, data from the study and control populations were assessed; however, there is a high risk of bias with this method of interpretation because the selective sampling method may have influenced pre‐test probability for the studied population.

**TABLE 2:**
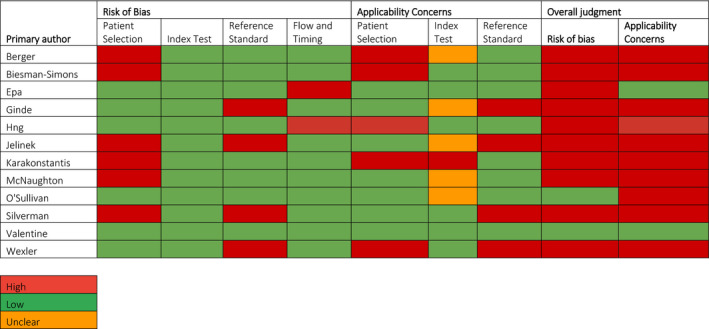
QUADAS‐2 assessment

All studies were considered to be at low risk of bias for the index test methodology, as all studies pre‐specified the blood glucose threshold above which participants were considered hyperglycaemic. We considered an HbA1c result ≥48 mmol/mol (≥6.5%) as an appropriate threshold for diabetes, as per the consensus in WHO^8^ and ADA^10^ guidelines. Four studies^26^, ^27^, ^28^, ^31^ using a diagnostic threshold lower than 48 mmol/mol (6.5%) were, therefore, considered at high risk of bias and limited in their applicability for the reference standard test. Two studies,^29^, ^30^ which followed up less than 80% of hyperglycaemic participants with HbA1c testing were considered at high risk of bias when assessed against the study flow and timing domain of QUADAS‐2.

Five studies^25^, ^27^, ^30^, ^33^, ^35^ were considered limited in applicability for patient selection due to cohort inclusions. These included study populations consisting only of elective surgical patients,^33^ or those selected using diabetes risk factors.^25^, ^35^ A proportion of the participants studied by Wexler et al.^27^ were admitted to ICU, so the study was considered to have a high degree of concern regarding applicability. Hng et al.^30^ was also considered to have limited applicability regarding patient selection as the inclusion criteria also included people aged between 16 and 18 years. Five studies^25^, ^28^, ^31^, ^34^, ^36^ using capillary rather than venous serum samples had unclear index test applicability because the extent to which a given capillary glucose measurement varies from a venous sample measurement for the same participant was unknown and not assessed. Biesman‐Simons et al.^33^ was considered of low concern for applicability as despite using capillary samples, the threshold for hyperglycaemia was adjusted to estimate a venous blood glucose value. Karakonstantis et al.^35^ was considered to be of high concern for applicability of the index testing used because blood glucose was measured in the morning in 46 of 55 screened participants and this was considered equivalent to a fasting glucose measurement.

### 3.2 Blood glucose testing for hospital patient populations

Extracted data for the index and diagnostic tests is shown in Table 3. We found considerable between‐study heterogeneity for the index testing protocols and results. Of 12 studies, seven measured blood glucose in ED,^25^, ^26^, ^28^, ^29^, ^30^, ^31^, ^36^ two used blood samples drawn as part of routine inpatient clinical care,^27^, ^32^ one used the first available morning blood glucose sample^35^ and one used a pre‐operative sample.^33^ One study did not specify when the sample was obtained.^34^ Five studies used a capillary sample,^25^, ^28^, ^31^, ^33^, ^36^ five used a venous sample,^26^, ^27^, ^29^, ^30^, ^32^ one used either^34^ (with two capillary or one venous blood glucose measurements over threshold sufficient to diagnose hyperglycaemia) and one did not specify the method of blood glucose sampling.^35^

**TABLE 3:** Index and diagnostic test data

Primary author	Index test – random blood glucose (RBG)									Diagnostic test – HbA1c		
	Diagnostic guidelines referenced for index and diagnostic test	Time during admission of index random blood glucose test	Capillary (finger prick) or venous serum sample for random blood glucose test	Context of HbA1c follow‐up (e.g. during admission, as an outpatient)	Random blood glucose threshold used to designate hyperglycaemic status (mmol/L)	Additional random blood glucose thresholds for sub‐classifying severity of hyperglycaemia (mmol/L)?	Threshold for diabetes diagnosis by HbA1c (mmol/mol, %)	Number of patients screened who had a random blood glucose test	Number of patients with glucose level above the predefined threshold (%)	Number who received reference diagnostic test Hba1c (% of patients with blood glucose above index test threshold)	Number with a diagnostic range HbA1c result (%)	Any further data assessing performance of the index test RBG (mmol/L) HbA1c (mmol/mol)
Berger	Unknown	In ED	Capillary	In ED	>11.1	n/a	>48 (>6.5)	332	11 (3.3%)	10 (90.9%)	9 (90%)	None
Biesman‐Simons	Society for Endocrinology Metabolism and Diabetes of South Africa (SEMDSA)	Preoperative	Capillary	Unknown	≥6.5	n/a	≥48 (≥6.5)	312	21 (6.7%)	19 (90.5%	5 (26.3%)	None
Epa*	Unknown	In ED	Venous	During admission	>7.8	>11.0, 7.9–11.0	≥48 (≥6.5)	16,268	844 (5.2%)	15 (7.5%)	3 (20%)	All three patients with HbA1c ≥48 (≥6.5) had RBG >11.0
Ginde	Unknown	In ED before IV fluids administered	Capillary (2 samples)	During admission	≥6.7	≥6.7, ≥7.2, ≥7.8	≥43 (≥6.1)	265	Unknown	265 (100%)	76 (28.7%)	Correlation between RBG and HbA1c r = 0.60 ≥6.7: 89% specificity and 26% sensitivity for HbA1c ≥48 (≥6.5) ≥7.2: 95% specificity, 18% sensitivity ≥7.8: 98% specificity, 14% sensitivity
Hng	RBG cut‐off: Valentine et al.²⁶ HbA1c: ADA	In ED	Venous	During admission	≥5.5	n/a	≥48 (≥6.5)	2,652	1,646 (62.1%)	1267 (77%)	157 (12.4%)	None
Jelinek	NHMRC, International Expert Committee recommendations, previous ED studies (Hewat et al.⁴⁵, George et al.⁴⁶)	In ED	Capillary	During admission (HbA1c at same time as RBG)	>6.0	n/a	>42 (>6.0)	590	198 (33.6%)	193 (97.5%	25 (11.9%)	Out of 584 HbA1c results, 13 patients had HbA1c >42 (>6.0) but RBG <6.0
Karakonstantis	ADA	First available morning blood glucose	Unknown	During admission	>7.0	5.6–7.0, >7.0	≥48 (≥6.5)	55	19 (34.5%)	19 (100%)	4 (21%)	27% of patients with BG >5.6 had HbA1c <39 (<5.7) 71% with BG 5.6–7.0 mmol/L had HbA1c 39–46 (5.7–6.4) 21% with BG >7.0 mmol/L had an HbA1c ≥48 (≥6.5) Agreement between BG <7.0 mmol/L and HbA1c <48 (<6.5) was 92%
McNaughton	ADA and Caribbean Health Research Council	In ED (HbA1c and RPG at same time)	Capillary	During admission	≥7.2	≥7.2, ≥8.6	≥48 (≥6.5)	228	Unknown	220 (90.5%)	9 (4.1%)	For HbA1c ≥48 (≥6.5): AUROC curve =0.94 (95% CI 0.91–0.97) RBG ≥7.2: 100% sensitive, 79% specific RBG ≥8.6: 67% sensitive, 92% specific
O'Sullivan	ADA	Unknown	Either	During admission	>10.0	n/a	≥48 (≥6.5)	262	126 (48.1%)	123 (97.6%)	11 (8.9%)	None
Silverman	RBG: ADA. HbA1c: Third National Health and Nutrition Examination Survey outpatient screening data	In ED before therapeutic intervention	Venous	During admission ‐ HbA1c from ED blood sample	>6.1	5.6–6.0, 6.1–7.0, 7.0–11.0, ≥11.1	≥44 (≥6.2)	541	331 (61.2%)	331 (100%)	74 (22.4%)	4.6% of patients with RBG ≤5.6 had HbA1c ≥44 (≥6.2) 7.6% with RBG <6.1 had HbA1c ≥44 (≥6.2) 12.5% with RBG 5.6–6.0 had HbA1c ≥44 (≥6.2) 16.4% with RBG 6.1 −7.0 had HbA1c ≥44 (≥6.2) 22.5% with RBG 7.0–11.0 had HbA1c ≥44 (≥6.2) 84.6% with RBG ≥11.1 had HbA1c ≥44 (≥6.2)
Valentine	International Expert Committee Recommendation	First blood sample drawn during routine clinical care	Venous	First blood sample drawn during clinical care	≥5.5	≥5.5 and ≥11.1	≥48 (≥6.5)	3,873	2,360 (61.0%)	2,360 (100%)	262 (11.1%)	RPG ≥11.1 mmol/L sensitivity of 28%, specificity of 98% ROC curve ‐ 0.78, 95% CI 0.75–0.81
Wexler	Unknown	During routine care	Venous	During admission	≥11.1	n/a	>43 (>6.1)	609	Unknown	695	123 (17.8%)	AUROC for HbA1c >43 (>6.1) =0.6 Positive predictive value of RBG >11.1 52%, negative predictive value 87% (*p*=0.07) 21/123 patients with elevated HbA1c >43 (>6.1) had RBG >11.1

Abbreviation: n/a, not applicable.

* In this study, a sample of 200 hyperglycaemic participants without known diabetes were selected for further study. These participants were chosen at random from the group of 844 subjects with hyperglycaemia and no known diabetes diagnosis. Further details are provided in Table 3.

We identified no studies that applied the same index test threshold and reference test threshold. Two pairs of studies used the same index threshold for hyperglycaemia (≥5.5 mmol/L^30^, ^32^ and ≥11.1 mmol/L^25^, ^27^), meaning that across the 12 studies, there were 10 different primary blood glucose thresholds. Six studies^26^, ^28^, ^29^, ^32^, ^35^, ^36^ used multiple thresholds ranging from ≥5.5 mmol/L to ≥11.1 mmol/L to subclassify the severity of hyperglycaemia.

Nine studies reported the proportion of participants screened, with an above‐threshold blood glucose test. The proportion of participants in each of these studies with an above‐threshold blood glucose level ranged from 3.3% to 62.1%, with a median (Q_1_, Q_3_) of 34.5% (5.95%, 61.1%). Across these nine studies, a total of 24,885 participants received a random blood glucose test; 5,556 (22.3%) were above threshold and, therefore, hyperglycaemic. Three studies^27^, ^28^, ^36^ only reported Area Under the Receiver Operating Curve (AUROC) statistical analyses and/or specificity and sensitivity of blood glucose to predict an abnormal HbA1c. These are discussed below.

### 3.3 Predictive value of blood glucose measurements for HbA1c testing

The HbA1c testing protocols were more consistent between studies. All but one^33^ carried out HbA1c testing during admission rather than outpatient follow‐up, and three of these^26^, ^31^, ^32^ tested HbA1c on blood taken at the same time as the sample used for random glucose testing. Out of the 12 studies, seven used HbA1c ≥48 mmol/mol (≥6.5%) to diagnose diabetes,^29^, ^30^, ^32^, ^33^, ^34^, ^35^, ^36^ one used HbA1c >48 mmol/mol (>6.5%),^25^ and four used values that were lower than 48 mmol/mol (6.5%).^26^, ^27^, ^28^, ^31^ With the exception of one study^29^ where 7.5% of hyperglycaemic participants were followed up with HbA1c, HbA1c reference testing was consistently high; in 10 studies 90% or more hyperglycaemic participants were assessed for diabetes using HbA1c.^25^, ^26^, ^27^, ^28^, ^31^, ^32^, ^33^, ^34^, ^35^, ^36^ The proportion of study participants diagnosed with diabetes varied from 4.1%^36^ to 90%,^25^ with a median (Q_1_, Q_3_) of 18.9% (11.5%, 61.1%). All the included studies performed a single HbA1c measurement as their reference test for their cohorts.

Eight studies^26^, ^27^, ^28^, ^29^, ^31^, ^32^, ^35^, ^36^ presented further data assessing the performance of blood glucose testing in predicting an above‐threshold HbA1c (Table 3). Three studies presented AUROC values of 0.94,^36^ 0.78^32^ and 0.6.^27^ An AUROC value greater than 0.7 is generally considered to indicate an acceptable level of sensitivity and specificity, with values over 0.8 considered excellent.^37^ Sensitivity and specificity data reported by the studies pertained to different thresholds, so pooling data for an overall assessment was not possible. Specificity of the index test was typically high in those studies in which it was reported; McNaughton et al.^36^ reports specificity of 79% for blood glucose ≥7.2 mmol/L and 92% for blood glucose ≥8.6 mmol/L whilst Ginde^28^ and Valentine et al.^32^ report specificity values of 89% or above for four separate thresholds.

Two studies^26^, ^35^ reported similar findings when a blood glucose threshold of >7.0 mmol/L was applied. Karakonstantis et al.^35^ report that 21% of participants with a blood glucose >7.0 mmol/L had an HbA1c ≥48 mmol/mol (≥6.5%), compared with 8% of participants with a blood glucose <7.0 mmol/L. Silverman et al.^26^ report data following this pattern; 26.7% of participants with a blood glucose >7.0 mmol/L had an HbA1c ≥44 mmol/mol (≥6.2%) compared with 11.1% of participants with a blood glucose <7.0 mmol/L. These results support the conclusion hypothesis that a higher blood glucose is associated with an increased prevalence of an elevated HbA1c measurement. It is worth that noting whilst the two studies used different HbA1c thresholds to indicate diabetes (Silverman et al.^26^ use a threshold of 44 mmol/mol (6.2%), whilst Karakonstantis et al.^35^ use a threshold of 48 mmol/mol (6.5%)), both report similar findings.

Four studies (Berger,^25^ Silverman,^26^ Wexler^27^ and Valentine^32^) use a threshold of 11.1 mmol/L. Berger et al.^25^ and Silverman et al.^26^ report that a high proportion (90%^25^ and 84.6%,^26^ respectively), of patients at this level of glycaemia also have an abnormal HbA1c. Valentine et al.^32^ reports a specificity of 98% and sensitivity of 28% for a 11.1 mmol/L threshold in predicting HbA1c ≥48 mmol/mol (≥6.5%) and Wexler et al.^27^ found that a threshold of 11.1 mmol/L has a positive predictive value of 52% and a negative predictive value of 87%. Despite the use of the same blood glucose threshold, we cannot compare the data presented in these papers to evaluate the performance of a threshold of 11.1 mmol/L in predicting an abnormal HbA1c, because the performance metrics reported for this threshold varied.

Four included publications^26^, ^27^, ^28^, ^31^ were published prior to 2011 and, therefore, antedate the WHO recommendation that HbA1c be used to diagnose diabetes above a threshold of 48 mmol/mol. All four used HbA1c thresholds below 48 mmol/mol to indicate diabetes. A further two studies^32^, ^34^ recruited participants prior to 2011 but were published after 2011, and both use HbA1c thresholds as per WHO guidelines. Although published prior to 2011, the studies by Ginde et al.,^28^ Silverman et al.^26^ and Wexler et al.^27^ all state that they use DCCT/IFCC aligned HbA1c measurements.

## 4 DISCUSSION

### 4.1 Summary of evidence

This review aimed to assess whether evaluation of glycaemic status using random blood glucose testing for adult hospital admissions can reliably detect undiagnosed diabetes. We identified 12 relevant studies. The median proportion (Q_1_, Q_3_) of hospital patients identified as hyperglycaemic was 34.5% (5.95%, 61.1%) and ranged from 3.3%^25^ to 62.1%.^30^ Thresholds defining hyperglycaemia ranged from 5.5 mmol/L to 11.1 mmol/L. The proportion of hyperglycaemic participants found to have a diabetes‐range HbA1c varied from 4.1%^36^ to 90%.^25^ All studies identified a proportion of their cohort as having a diabetes‐range HbA1c, indicating the potential value of screening for diabetes during hospitalisation.

Clinical investigation of inpatient hyperglycaemia is infrequent,^38^, ^39^ despite high prevalence^40^ and physician support for increased intervention.^41^ There appears to be a lack of clinical research in this area, with only a small number of studies identified in this review. This could be attributed to limited resources, the challenging nature of identifying and acting on those identified at risk, a lack of consistent management guidelines, and, importantly, the assumption that in‐hospital hyperglycaemia is attributable to a stress response rather than indicative of underlying diabetes. This review has signalled that hyperglycaemia in the acute and inpatient hospital settings can be indicative of underlying diabetes. It highlights the prevalence of in‐hospital hyperglycaemia and demonstrates that with all index test thresholds and reference test thresholds that have been deployed in clinical studies to date, a clinically relevant proportion of participants with hyperglycaemia have been found to have a diabetes‐range HbA1c. Therefore, in‐hospital blood glucose testing, particularly where automated systems can be utilised, may provide a window of opportunity for earlier detection of undiagnosed diabetes.

### 4.2 Strengths and limitations at study and outcome level

The main limitations are highlighted by the heterogeneity between included studies, which is reflected in the QUADAS‐2 assessment. Only one study was low risk for both bias and applicability,^32^ while a further two were low risk for only bias^34^ or applicability.^29^, ^30^ The majority of study protocols only tested HbA1c for participants with an above‐threshold blood glucose measurement, so authors did not report the number of participants below threshold and did not assess the HbA1c in these individuals. Therefore, for the majority of studies, the number of participants with diabetes with a below‐threshold random blood glucose is not known and it was not possible to calculate specificity and sensitivity for the thresholds used in these studies.

There was considerable between‐study variation in the proportion of study participants with an above‐threshold blood glucose and the proportion who were subsequently diagnosed with diabetes. Possible sources of heterogeneity accounting for this include the variety of index and diagnostic test thresholds applied, the use of capillary samples for blood glucose testing (where hospital‐based quality assurance methods were not specified), cohort demographics, variation in participant eligibility, hospital setting, and sampling strategies deployed. Wexler et al.^27^ did not exclude patients with known diabetes during the recruitment stage of their trial. However, the authors report data separately for those individuals with previously detected diabetes and previously undetected diabetes. The data that we have extracted for this review do not include participants with previously detected diabetes.

Study nation varied across the studies included in this review, which may contribute to variation in the study outcomes as HbA1c has been found to underestimate average blood glucose in African‐Americans populations with sickle cell trait^42^ and higher HbA1c values have been reported in African‐Americans and American Indians compared with white Americans, independent of haemoglobin variants.^43^

Only one study (Karakonstantis et al.^35^) excluded individuals with medical conditions, which might affect HbA1c accuracy. HbA1c can underestimate glycaemia in HIV‐infected^44^ and G6PD‐deficient^45^ individuals, and overestimate glycaemia in people with iron‐deficiency anaemia.^43^ Corticosteroid use can cause persistent hyperglycaemia (‘steroid diabetes’)^46^ so its effect on HbA1c should be considered, especially in a hospital patient population, although literature on the subject is scarce. The possibility that illness preceding hospital admission, including acute pancreatic damage or renal failure, could perturb HbA1c should also be considered as a possible limitation.^47^ Both NICE and the ADA recommend cautious interpretation of HbA1c and suggest oral glucose tolerance testing or fasting blood glucose testing be used for individuals with conditions that might affect HbA1c.^7^, ^10^ This should be incorporated in future study designs and clinical judgment exercised in considering the possibility of conditions that may alter HbA1c.

Variation in thresholds used to define hyperglycaemia and HbA1c among the identified studies prevented an overall assessment of the diagnostic performance of the thresholds applied. In addition, comparison of study results was hindered because the reporting of findings differed between studies, with each using different measures of diagnostic performance. WHO guidelines state that a fasting blood glucose ≥7.0 mmol/L is diagnostic of diabetes,^6^ so it is perhaps surprising that six reviewed studies use thresholds lower than this for random blood glucose testing.^26^, ^28^, ^30^, ^31^, ^32^, ^33^ Two of these based their thresholds on previous publications; Hng et al.^30^ cite Valentine et al.,^32^ and Jelinek et al.^31^ cite Hewat et al.^48^ and George et al.,^49^ both of which were deemed ineligible for inclusion in this review because neither used HbA1c for reference diagnostic testing.

Eight included studies^25^, ^29^, ^30^, ^31^, ^32^, ^33^, ^34^, ^36^ do not explicitly state how HbA1c measurements were standardised. However, of these, three^30^, ^34^, ^36^ cite ADA diagnostic guidelines, which state that a ‘HbA1c test should be performed in a laboratory using a method that is NGSP certified and standardized to the DCCT assay’.^10^ Studies that do not use a standardised assay for HbA1c measurements, or which use point of care HbA1c testing, may not be generalizable beyond the study setting.

WHO guidelines require two diagnostic‐threshold HbA1c results to confirm a diagnosis of diabetes.^8^ All studies identified use a single HbA1c measurement as indicative of diabetes, and future study design should incorporate follow‐up testing in the community to confirm a diagnosis.

### 4.3 Strengths and limitations at review level

Strengths of this review include the systematic approach taken for study identification and data extraction, as per the registered PROSPERO protocol, and its concordance with the PRISMA‐DTA Statement. Our search strategy was developed with a medical information specialist, no study nations or language restrictions were applied, and grey literature was searched. The authors of grey literature citations, which were deemed potentially eligible, were contacted for further information and data, with some success, and a consistent approach was taken for screening, data extraction and quality assessment, involving two independent reviewers and adjudication by a third.

In two instances, we have included studies in which part of the study cohort does not meet our inclusion criteria. Hng et al. included participants aged 16 and above, whereas Wexler include a subset of patients admitted to ICU. We have included these studies to ensure that we summarise all existing data regarding the ability of random blood glucose to predict an abnormal HbA1c. However, these studies should be interpreted with caution, and this is reflected in our QUADAS‐2 assessment.

Studies using a diagnostic test other than HbA1c (e.g. fasting blood glucose and OGTT) were excluded. Although this could be seen as a limitation because these excluded studies still assessed the performance of blood glucose in diabetes screening, we believe this decision was clinically appropriate for several reasons. Firstly, HbA1c is less likely to be affected by stress hyperglycaemia^50^ because it reflects longer‐term glycaemic control and can differentiate between acute hyperglycaemia and an underlying metabolic deficiency. Secondly, HbA1c testing can be safely and universally performed in hospital patient populations as opposed to fasting blood glucose or OGTT because HbA1c does not require people to fast or undergo glucose loading.

### 4.4 Implications for future research and clinical practice

Questions remain as to the most appropriate random blood glucose threshold for identifying a level of hyperglycaemia that can indicates a need for subsequent diagnostic testing. This highlights a need for further high‐quality research in this field, ideally reported according to international guidelines for the reporting of diagnostic accuracy studies such as STARD.^51^ For consistency, future study protocols should, if possible, test blood glucose with venous, rather than capillary samples, as capillary glucose has been found to be higher than venous glucose within the same individual: Studies have reported differences from 0.58 mmol/L^52^ to 2.8 mmol/L,^53^ and the difference can vary depending on when samples are taken.^54^ In addition, further data on time lag before measurement in the laboratory, within study validation of point of care measurements, and standardised quality assurance should be provided. Where resources are constrained and capillary testing essential, capillary measurements should be adjusted as per Society for Endocrinology, Metabolism and Diabetes of South Africa (SEMDSA) guidelines.^55^

Future research identifying a random blood glucose threshold that indicates a high risk for diabetes could have wide‐reaching benefit, by facilitating the implementation of a cost‐effective in‐hospital screening strategy for diabetes and non‐diabetic hyperglycaemia. This could aid existing efforts coordinated by the NHS Diabetes Prevention Programme to decrease the prevalence of diabetes and improve diabetes diagnostics in the United Kingdom.

## 5 CONCLUSIONS

This review has demonstrated that when hospital patients are stratified for HbA1c testing using a random blood glucose test, this will consistently detect a proportion of patients with diabetes‐range HbA1c. Hyperglycaemia in hospital, therefore, cannot solely be attributed to an acute stress response. Hospital attendance could be considered as a window of opportunity in which to assess individuals who may otherwise not receive a diabetes diagnosis until they develop symptoms, at which point most will have irreversible end‐organ damage. The proportion of participants with hyperglycaemia and the proportion of hyperglycaemic participants found to have a diabetes‐range HbA1c varied between the included studies, indicating the need for consistency in defining in‐hospital hyperglycaemia and highlighting the importance of further rigorous research in this field.

## CONFLICT OF INTEREST

AJF is Director of the NIHR Health Technology Assessment Programme and receives funding support from the NIHR Oxford Biomedical Research Centre. This research was funded in whole or in part by the Wellcome Trust [203921/Z/16/Z]. For the purpose of Open Access, the author has applied a CC BY public copyright license to any Author Accepted Manuscript (AAM) version arising from this submission.

## APPENDIX 1. Search terms

Searches were run on 3 November 2020 and rerun on 11 January 2021.

**PubMed** : No country, language or date restrictions. Search terms were as follows:

in‐hospital OR hospitali?ed OR inpatient OR “emergency department”

AND

hyperglyc?emia OR "raised glucose” OR glucose

AND

"formal assessment" OR diagnosis OR follow‐up OR outpatient OR community

AND

"diabetes mellitus" OR HbA1c OR “glycated haemoglobin" OR "haemoglobin A1c" OR “glycated hemoglobin” OR “hemoglobin A1c”

AND

undiagnosed OR “no prior history" OR asymptomatic OR "without known diabetes" OR "without a diagnosis"

**Embase** : No country, language or date restrictions. Each line was searched using <term>.mp, where mp=title, abstract, heading word, drug trade name, original title, device manufacturer, drug manufacturer, device trade name, keyword, floating subheading word, candidate term word. Search terms were as follows:

in‐hospital OR hospitali?ed OR inpatient? OR “emergency department”

AND

hyperglyc?emia OR "raised glucose” OR glucose

AND

"formal assessment" OR diagnosis OR follow‐up OR outpatient? OR community

AND

"diabetes mellitus" OR HbA1c OR “glycated h?emoglobin" OR "H?emoglobin A1c"

AND

undiagnosed OR “no prior history" OR asymptomatic OR "without known diabetes" OR "without a diagnosis"
